# The effect of atopic dermatitis in pediatric patients on height: Reflections triggered by a real-life case report

**DOI:** 10.1097/MD.0000000000036150

**Published:** 2023-11-24

**Authors:** Mei Zhang, Ming He, Ting Tang

**Affiliations:** a Guizhou University of Traditional Chinese Medicine, Guiyang, China; b Department of Dermatology, The First Affiliated Hospital of Guizhou University of Traditional Chinese Medicine, Guiyang, China.

**Keywords:** atopic dermatitis (AD), case report, difference, height, twins

## Abstract

**Rationale:**

Atopic dermatitis (AD) is a burdensome skin disorder, especially in children. The prevalence of children with AD is increasing year by year in China. Typical symptoms like eczema-like lesions and severe pruritus can seriously affect the sleep quality and the growth and development of pediatric patients.

**Patient concerns:**

We observed a pair of fraternal twins, in which the younger sister had AD, while the elder one had no relevant medical history. At present, the height of the 2 individuals is significantly inconsistent, with a difference of about 10 cm.

**Diagnoses:**

Based on the little patient’s medical history and clinical manifestations, the diagnosis of AD was made.

**Interventions:**

This little patient was treated with oral routine antihistamines, topical glucocorticoids and Dupilumab.

**Outcomes:**

At present, her rash and xerosis have significant improvement. She also have relief of generalized itching and improved sleep quality.

**Lessons:**

Previous studies have indeed shown that AD has a negative impact on children’s height. This case leads us to consider the association between AD and height. It also gave us the opportunity to observe subsequent height changes after the intervention was carried out.

## 1. Introduction

Atopic dermatitis (AD) is a chronic, recurrent, and inflammatory skin disease to some extent associated with short stature in AD children and the causes of the impaired growth in them are still unknown. It is believed that the mechanism is multifactorial, like sleep disturbance, inappropriate exclusion diets, and so on. At the same time, the results of previous studies suggest that growth impairment from AD in childhood is likely to be temporary. To verify the viewpoints above, we will follow up a pair of twins in which younger sister has AD and there is already difference in height between them.

## 2. Case report

**Figure 1.**
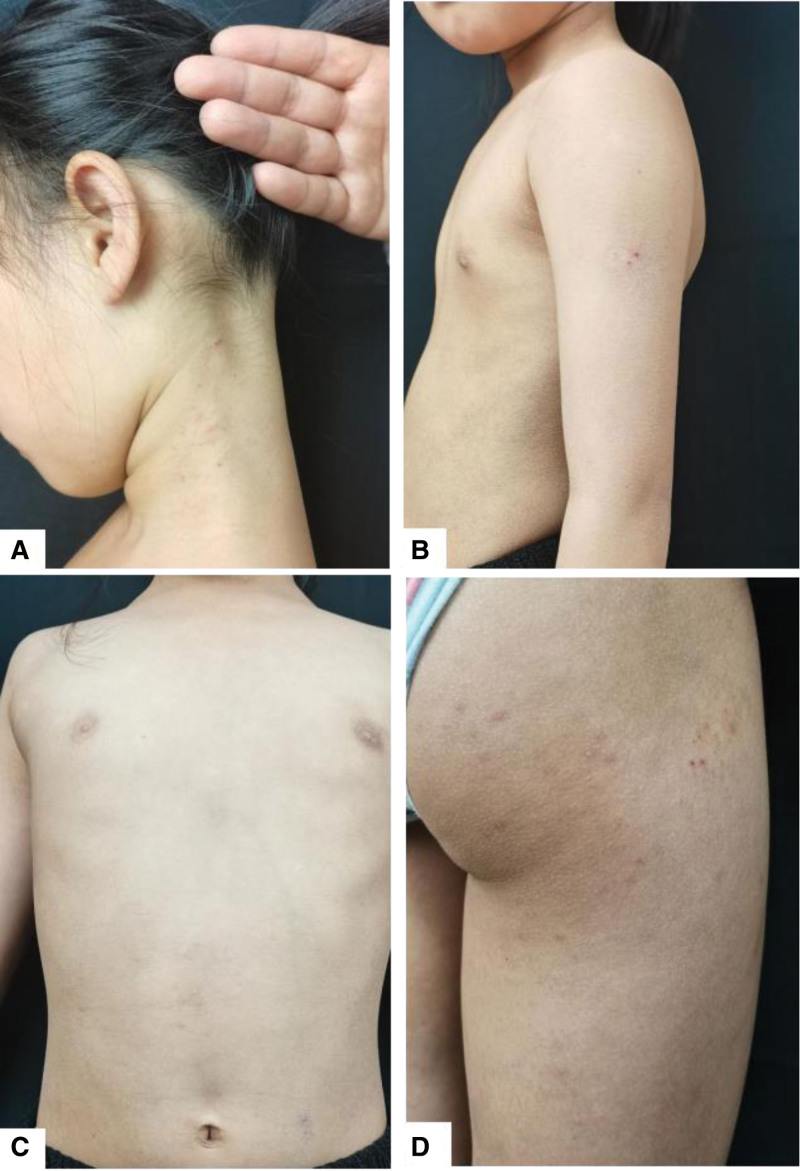
(A–F) Dry skin, scattered papules, scratchs, and hemorrhagic crusts all over the body. Scattered hyperpigmentation of lesions after healing also can be seen. (G and H) Circumscribed erythematous plaques in the medial side of both foot arches. Approximately 10% total body surface area involvement.

On February 12, 2023, a 7-year-old little female patient led by her parents came to our consulting room for the first time with dry skin, scattered papules, scratchs, and hemorrhagic crusts all over the body. Circumscribed erythematous plaques in the medial side of both foot arches. Scattered hyperpigmentation of lesions after healing also can be seen (Fig. 1). Approximately 10% total body surface area involvement. Her blood routine result was normal and allergen testing showed total IgE +++. Her mother has been suffering from allergic rhinitis.

Her parents told us that 4 years ago, dry skin and scattered papules occurred all over the body without obvious cause in younger sister. She had significant itching as well. Since then, she was continuously diagnosed with eczema and accepted various conventional therapies such as oral antihistamines, topical glucocorticoids, Chinese herbal ointment, Chinese medicinal bath, and so on. The efficacy of these treatments were not satisfactory; her condition has not improved significantly to date.

**Figure 2.**
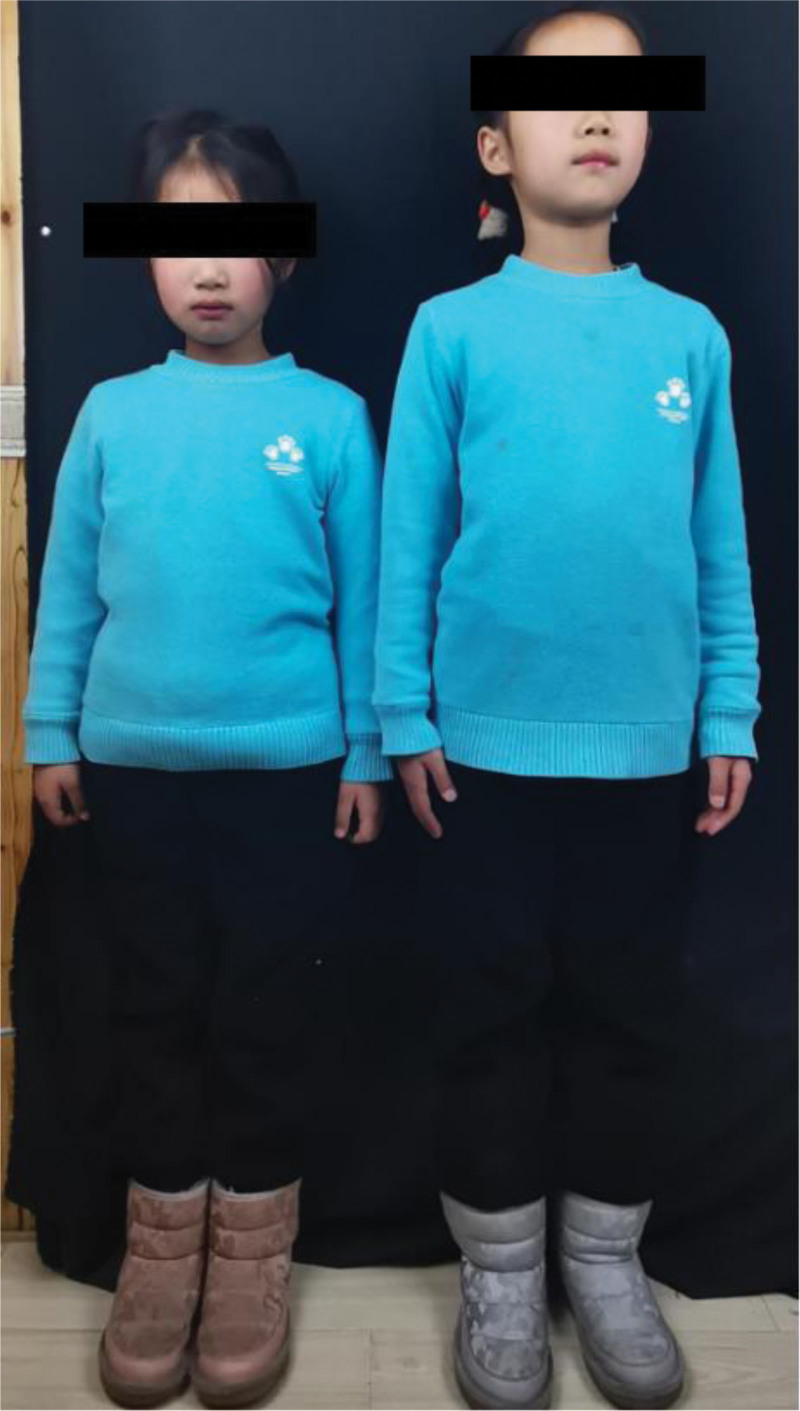
The difference in height between our little patient and her elder sister.

By this point you may think that this is just a typical case of misdiagnosed pediatric AD with nothing special about it. What is not, however, is that the child’s father mentioned that the difference in height between she and her twin elder sister was surprisingly nearly 10 cm. Moreover, the height difference was gradually revealed after the onset of the child’s illness 4 years ago. We can clearly see the difference when the twins stand together (Fig. 2).

**Figure 3.**
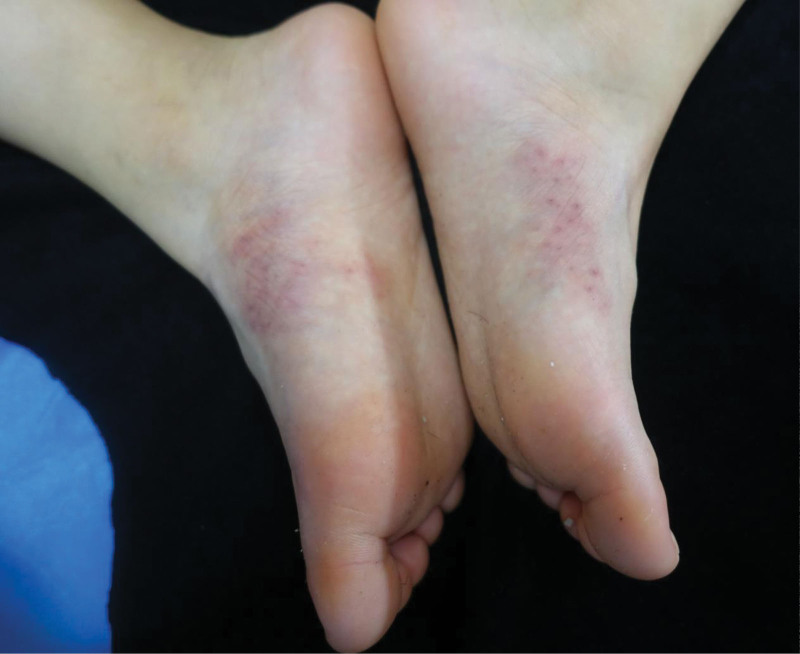
Skin lesions in the medial side of both foot arches after treatment.

On the day of the first visit, the patient received her first dose of 600 mg Dupilumab, subcutaneously. The loading dose was followed by 300 mg every 4 weeks. Dupilumab is a fully human monoclonal antibody that is directed against the interleukin-4α receptor, thereby blocking both interleukin-4 and interleukin-13 signaling and hence type 2 inflammation.^[1]^ It has been approved for the treatment of moderate-to-severe AD.^[2]^ On May 3, 2023, she received the 4th cycle of Dupilumab. At present, her rash and xerosis have significant improvement (Fig. 3). She also have relief of generalized itching and improved sleep quality.

## 3. Discussion

In a 2022 large cohort study of 10,611 children,^[3]^ researchers found that AD was associated with shorter stature among young children. Though there are conflicting data available, a study published in JAMA which using 9 US population-based cross-sectional studies^[4]^ found no significant difference in height between children with AD and children without AD, previous studies did suggested AD has a negative effect on children’s height.^[5–8]^

Growth and development are very complex processes which are easily disturbed. The causes of the impaired growth in AD children are still unknown. Almost all studies agreed that the mechanism is multifactorial. Sleep disturbance, which characterized with decreased sleep duration, low sleep efficiency, and increased awakenings, had considered to be the biggest impact on shorter stature in children with AD.^[9]^ The more severe the disease, the worse the quality of sleep.^[10]^ Growth hormone (GH) is closely related to height growth in children; it peaks immediately subsequent to sleep onset.^[11]^ Therefore, sleep disturbance can affect the secretion of GH, resulting in stunted growth and development.

Inappropriate exclusion diets is another notable cause related to shorter stature in AD children.^[6]^ Dietary modification is a fundamental treatment in AD, but parents were highly influenced by the results of allergen testing to perform strict food restriction. It is worth mentioning that this phenomenon is very common in remote areas of China. Food-restricted pediatric patients have lower calories, protein, carbohydrate, fat, riboflavin, vitamin B12, phosphorus, calcium, and iron intakes, which are all essential for growth.^[12]^

Long-term use of corticosteroids should not be excluded as a potential cause of growth impairment. Children with chronic diseases usually receive prolonged systemic corticosteroids treatment, as this is well recognized to be contributed to shorter stature.^[13]^ But systemic corticosteroids are rarely used in pediatric AD patients, as topical corticosteroids (TCSs) are first-line therapy. An umbrella review found^[14]^ no evidence of harm when TCSs were used intermittently as required. However, long-term safety data were limited. On the other hand, adequate control of AD throughout childhood with appropriate use of TCSs may prevent the need for systemic or higher-potency TCSs and may mitigate any potential effects on growth.^[3]^

Back to our case in this article, according to her parents, our little patient had not been on a strict diet, and had rarely used TCSs for 1 consecutive week because of fear of side effects since she suffered from AD, let alone systemic glucocorticoids. Much more meaningful is that her sleep has been continuously and obviously affected at night due to pruritus. Specifically in low sleep efficiency and increased awakenings. Unfortunately, our little patient was unable to measure GH levels due to family refusal. During the first consultation, she acted anxiously and irritably. Her parents told us that she has no decreased productivity at school, and without detrimental effects on emotional and social life, but minded the comparison of height between she and her elder sister.

For the association between AD and decreased height, it was not static as children aged, which would attenuated by age 14 years.^[3]^ A tendency for catch-up growth was observed, and with correction by adolescence. For this phenomenon, we consider the following reasons: (a) 70% affected children will experience significant improvement in symptoms later in childhood.^[15]^ (b) After the occurrence of AD, most of pediatric patient would be treated aggressively and reasonably before adolescence.

Just as mentioned above, there is a tendency for catch-up growth in AD children. The patient will be followed up continuously, with special attention to her growth and development. There are also several thought-provoking questions.

The delayed bone age is probably an associated feature of growth impairment, since the children with growth delay appeared to have a bone age retardation to a similar degree.^[5]^ Skeletal retardation is related to abnormal bone metabolism, so how do the pathophysiological basal mechanisms of AD affect bone metabolism in AD children? By what series of reactions? Future researches in this field are promising.

These 2 girls are fraternal twins. There are differences in genetic information. Although there is no obvious difference in the height of them 4 years ago, whether the difference in height is influenced by genes to some extent? We still unknown. A series of follow-up studies including genetic testing are needed.

## 4. Conclusion

With this case, we delved into the association between AD and height. Based on the previous literature, the results were positive. Whether AD actually has an effect on the height of our little patient or whether it is related to the fact that the 2 are fraternal twins needs to be followed up.

## Acknowledgments

The authors would like to thank the patient and her family who are willing to share her case. The authors would also like to thank the physicians, nurses, research coordinators, and other staff at the hospital.

## Author contributions

**Supervision:** Ming He, Ting Tang.

**Writing – original draft:** Mei Zhang.

**Writing – review & editing:** Ting Tang.
